# Temperature- and pH-responsive injectable chitosan hydrogels loaded with doxorubicin and curcumin as long-lasting release platforms for the treatment of solid tumors

**DOI:** 10.3389/fbioe.2022.1043939

**Published:** 2022-11-03

**Authors:** Na Li, Jianjun Lin, Chunping Liu, Qian Zhang, Riwang Li, Chuang Wang, Chaochao Zhao, Lu Lu, Changren Zhou, Jinhuan Tian, Shan Ding

**Affiliations:** ^1^ Foshan Stomatology Hospital, School of Medicine, Foshan University, Foshan, China; ^2^ Engineering Research Center of Artificial Organs and Materials, Guangzhou, China; ^3^ Department of Materials Science and Engineering, Jinan University, Guangzhou, China

**Keywords:** hydrogel, pH-sensitive, injectable, curcumin, doxorubicin, liposome, solid tumor

## Abstract

The efficacy of treating solid tumors with chemotherapy is primarily hindered by dose-limiting toxicity due to off-target effects and the heterogeneous drug distribution caused by the dense extracellular matrix. The enhanced permeability and retention (EPR) effect within tumors restricts the circulation and diffusion of drugs. To overcome these obstacles, hydrogels formed *in situ* at the tumor site have been proposed to promote drug accumulation, retention, and long-lasting release. We developed a thiolated chitosan (CSSH) hydrogel with a gelation point of 37°C. Due to the pH-sensitive characteristics of disulfides, the prepared hydrogel facilitated drug release in the acidic tumor environment. A drug release system composed of hydrophilic doxorubicin (Dox) and hydrophobic liposome-encapsulated curcumin (Cur–Lip) was designed to enhance the long-lasting therapeutic impacts and reduce adverse side effects. These composite gels possess a suitable gelation time of approximately 8–12 min under physiological conditions. The cumulative release ratio was higher at pH = 5.5 than at pH = 7.4 over the first 24 h, during which approximately 10% of the Dox was released, and Cur was released slowly over the following 24–120 h. Cell assays indicated that the Cur–Lip/Dox/CSSH gels effectively inhibited the growth of cancer cells. These *in situ*-formed Cur–Lip/Dox gels with long-term drug release capabilities have potential applications for tumor suppression and tissue regeneration after surgical tumor resection.

## 1 Introduction

Cancer is the second most frequent cause of death worldwide. Solid tumors, such as lung, colon, prostate, and breast cancer, are the major culprits of cancer mortality (Titov et al., 2020). Therapies, including radiotherapy, chemotherapy, and the surgical removal of tumors, are used for cancer treatment in clinics (Lang et al., 2019; Mirza and Karim, 2019). These methods exhibit good therapeutic effects in the initial treatment stage, but serious side effects and drawbacks in subsequent stages, such as irreversible damage to the patient’s body, resistance to radiation, and a high risk of recurrence, cannot be ignored (Ma et al., 2019; Martin et al., 2019). Adoptive immunotherapy is a promising therapeutic modality, especially chimeric antigen receptor (CAR) T-cell therapy. However, solid tumors engage numerous mechanisms that disrupt acquired immunity, which restrict the clinical performance of adoptive immunotherapy. To overcome this dilemma, the use of photodynamic therapy (PDT) has been suggested due to its low toxicity. However, the low solubility of photosensitizers (PSs) in aqueous environments restricts their effective administration in blood circulation and specificity to combine with tumors. Therefore, chemotherapy repurposed to induce antitumor immune responses by water-soluble PSs has the potential for high cancer killing performance (Mei et al., 2020).

Although chemotherapy has been successful and encouraging, most chemotherapy regimens involve the systemic administration of cytotoxic drugs that are often associated with dose-limiting toxicity due to off-target effects (Abyaneh et al., 2020). The enhanced permeability and retention (EPR) effect caused by a leaky vasculature and impaired lymphatic drainage within the tumor reduce the circulation and diffusion of drugs. Therefore, improving drug retention in tumors and reducing side effects are major challenges. Notably, the use of a long-lasting *in situ* release platform with multiple functions is one approach to resolve these problems.

Hydrogels have a three-dimensional network and are capable of encapsulating other compounds to facilitate the delivery and transport of drugs, nutrients, metabolites, and necessary additives. Injectable hydrogels are worthy of in-depth study due to their ability to rapidly form *in situ* and fill defects or coat objects with irregular shapes and sizes (Kim et al., 2017; Albashari et al., 2020; Wang et al., 2020; Luo et al., 2022). Our earlier work reported that thermosensitive thiol-derivatized chitosan (CSSH) underwent a rapid sol–gel transition under physiological conditions due to the formation of disulfide bonds, hydrogen bonds, and hydrophobic interactions (Ye et al., 2016; Li et al., 2017; Ye et al., 2018; Lin et al., 2020). The abundant amino and thiol groups of CSSH are also sensitive to the low pH of the tumor environment and promote the targeted release of antitumor drugs. The excision of breast or other tissues because of tumors causes great psychological stress and inconvenience to patients (Finak et al., 2008; Waks and Winer, 2019), and high tumor recurrence threatens the patient’s life. Therefore, biocompatible and biodegradable CSSH hydrogels are suitable drug release platforms to treat solid tumors *in situ* and prevent tumor recurrence after excision.

Curcumin (Cur) is extracted from ginger rhizomes and contains unsaturated aliphatic and aromatic groups (Rafiee et al., 2019). As a widely used drug, it exhibits good biocompatibility and degradability, low toxicity, and few side effects in the human body. Cur also inhibits the proliferation of cancer cells (Choudhuri et al., 2002; Tang et al., 2010;Liu et al., 2019;Raveendran et al., 2016; Sun et al., 2017; Li et al., 2018). However, its poor water solubility and low bioavailability limit its clinical applications. Liposomes (Lips) encapsulate hydrophobic drugs to improve their water solubility. The phospholipid bilayer structure of Lips is similar to that of the cell membrane, which favors the fusion of cancer cell membranes and Lips to promote drug delivery (Zhang et al., 2018; Cheng et al., 2020). Therefore, the water solubility of Cur may be efficiently improved by encapsulating it into Lips (Cur/Lips). However, Lips are a colloidal system with weak intermolecular forces and thermal stability. We propose a method for the formation of a CSSH hydrogel system by coating Cur/Lips with CSSH to improve the stability of Cur.

Hydrophilic doxorubicin hydrochloride (Dox) is a broad-spectrum anticancer drug. Its underlying mechanism of action is disruption of double-stranded DNA, which causes repeated DNA damage and inhibits tumor cell proliferation. However, cancer cells easily develop resistance to Dox. Long-term Dox use also causes strong cardiotoxicity, which primarily manifests as congestive heart failure (Tap et al., 2016; Cappetta et al., 2018; Lovitt et al., 2018). Encapsulating Cur and Dox into colloidal particles significantly reverses the drug resistance of cells (Gou et al., 2011; Zhu et al., 2016). Other controlled drug release systems, such as nanoparticles, capsules, and microgels made from natural or synthetic polymers or inorganic materials have also been widely studied (Wang et al., 2006; Chen et al., 2014; Yang et al., 2017). However, our earlier studies demonstrated that CSSH hydrogels had excellent biological and physicochemical properties. Therefore, the application of CSSH gels as Dox release carriers should be a good choice.

**FIGURE 1:**
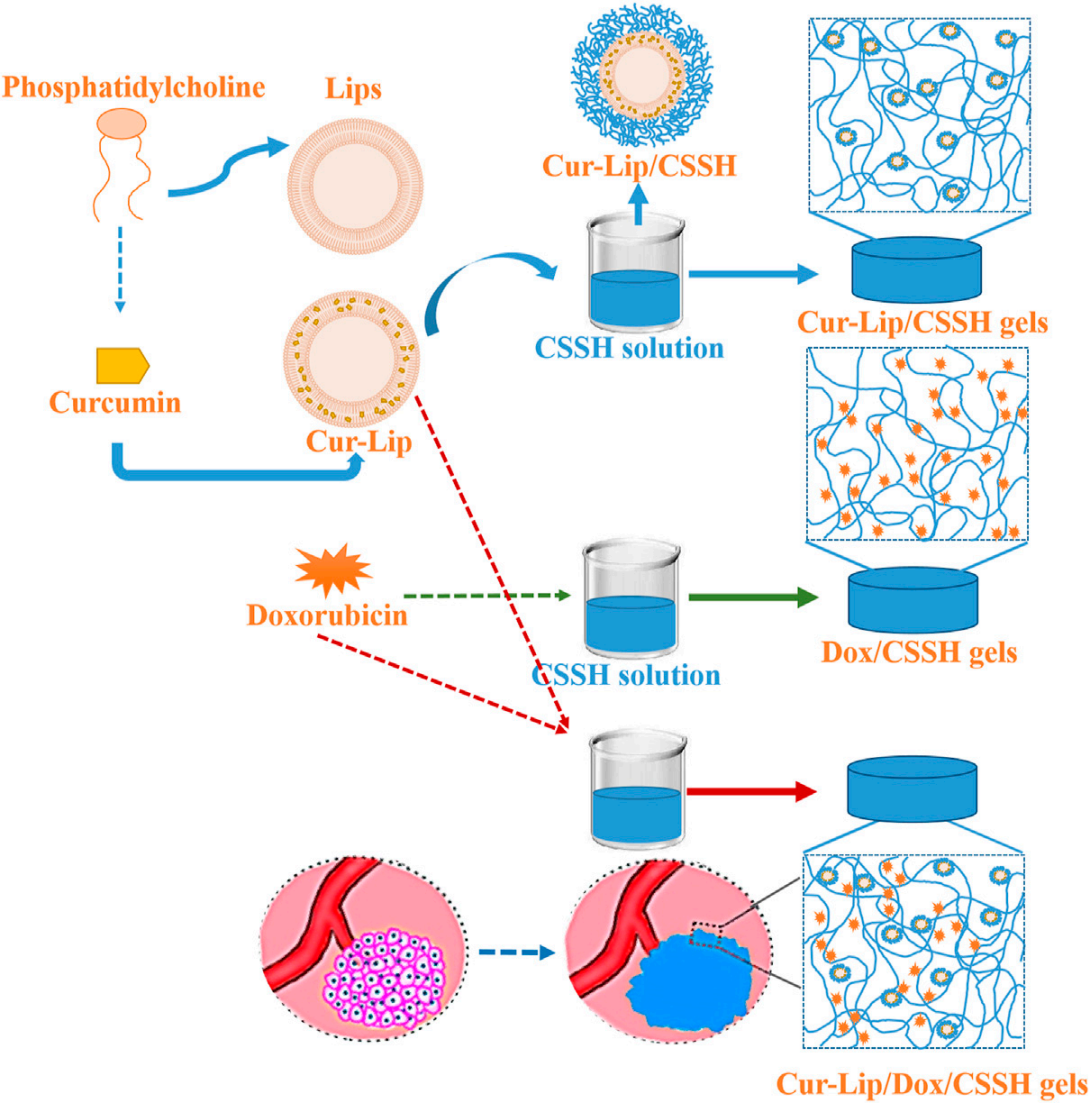
Schematic illustration of the *in situ-*injected double drug release system for solid tumor treatment.

The present study designed a smart, temperature-responsive CSSH hydrogel to facilitate the *in situ* coating of solid tumors, repair the leaky vasculature and impaired lymphatic drainage, and fill defects after tumor excision. The pH-sensitive disulfide bonds of these hydrogels enable drug release in the acidic tumor microenvironment. This platform may be used for local immobilization and sustained release of drugs to inhibit the proliferation of tumor cells (Figure 1).

## 2 Materials and methods

### 2.1 Materials

Chitosan (CS, medium molecular weight, deacetylation degree = 75∼85%), L-cysteine hydrochloride monohydrate (Cys), L-alpha-phosphatidylcholine 14–23% choline basis (PC), and cholesterol were purchased from Sigma‒Aldrich. Analytical grade N-(3-dimethylaminopropyl)-N′-ethylcarbodiimide hydrochloride (EDAC) and N-hydroxysuccinimide (NHS) were purchased from Qi Yun Biotechnology Company (Guangzhou, China). Dox and Cur were obtained from Shuoheng Biotechnology Company (Guangzhou, China). MCF-7 cells were purchased from American Type Culture Collection (ATCC, CRL2593).

### 2.2 Synthesis of Dox/Cur–Lip hydrogels

#### 2.2.1 Preparation of CSSH

CS powder was dissolved in a 0.5% (vol/%) glacial acetic acid solution, stirred overnight, and filtered to a final concentration of 1.0% (wt%), as described previously with modifications (Ding et al., 2012; Ye et al., 2016; Ye et al., 2018). EDAC and NHS were added for 30 min for functional group activation, and a stoichiometric amount of Cys was added to introduce the thiol group into the CS molecular chain. NaOH (1 M) was used to adjust the pH to 5–6, and the reaction was allowed to proceed for 5 h in the dark. The products were dialyzed using a dialysis bag (8,000–14000 Da) for 3 days in deionized water, pure water containing 1% (m/v) NaCl, and deionized water in succession. All dialysates were adjusted to pH = 5∼6 by addition of 1 M HCl solution. The obtained CSSH product was freeze-dried for subsequent use.

#### 2.2.2 Lip preparation and modification

Lips loaded with and without Cur were prepared using the thin-film hydration method (Dua et al., 2012; Li et al., 2017). Briefly, PC, cholesterol, and Cur were dissolved in ethanol at a mass ratio of 15:3:1. After evaporation of the solvent, the resulting lipid film was hydrated with 30 ml of deionized water to generate Cur–Lips. The final concentrations of PC, cholesterol, and curcumin were 10 mg/ml, 2 mg/ml, and 1 mg/ml, respectively. The Lip suspensions were homogenized using an ultrasound cell breaker (power: 30%, 3 s on, 3 s off).

CSSH (0.3 mg/ml) was dissolved in 10 ml of deionized water. A Lip suspension (10 ml) was slowly added to the polymer dispersion under stirring for 1 h to obtain the final coated vesicles (Zhuang et al., 2010). The coated vesicle dispersions were ultracentrifuged at 15,000 r/min for 1 h at 4°C to remove the excess polymer, followed by three washes with distilled water. The final products were labeled Lip-CSSH and Cur–Lip-CSSH.

#### 2.2.3 Preparation of single/double drug-loaded hydrogels

CSSH was dissolved in solution (pH = 8.0). β-Glycerol phosphate (β-GP) was added to the CSSH solution at a mass-to-volume ratio of 29%. The gel precursor solution was obtained when the mixture became homogeneous. The solution was placed into a 1-ml well and stored at 37°C to form a gel. Cur–Lip CSSH gels, Dox CSSH gels, and Cur–Lip/Dox CSSH gels were prepared using the protocol with the addition of Cur–Lip and/or Dox before β-GP.

### 2.3 Characterization

#### 2.3.1 Characterization of CSSH

The presence of the SH group was confirmed using Fourier transform infrared (FT-IR) spectroscopy (Quinox 55, Germany) and proton nuclear magnetic resonance (^1^H NMR) spectroscopy (AVANCE III 500, Germany). Briefly, samples mixed with KBr were pressed into a flaky shape. Total reflection scanning (*λ* = 4,000–600 cm^−1^) was performed on an FT-IR instrument. Five milligrams of each of CS and CSSH was added to a centrifuge tube and mixed with 0.55 ml of D_2_O/CF_3_COOD (95:5 v/v) and 0.55 ml D_2_O. The resulting mixed solution was placed in an NMR tube for ^1^H NMR analysis (AVANCE III 500, Germany).

#### 2.3.2 Characterization of Lips and Cur–Lips

Samples were diluted with water and measured in triplicate. The nanoparticle size distribution and zeta potential were measured using a laser particle size analyzer (Malvern Instruments Ltd., United Kingdom) and the dynamic light scattering (DLS) technique.

The morphology of the Lips and Cur–Lips was observed using transmission electron microscopy (TEM, Philips Tecnai 10). The samples were prepared by placing diluted Lip, Lip-CSSH, Cur–Lip, and Cur–Lip/CSSH solutions on a copper grid, followed by staining with 2% phosphotungstic acid for 1 min and drying naturally.

Unencapsulated Cur was removed by Sephadex G-25 *via* microcolumn centrifugation (Sun et al., 2017). Cur–Lips and Cur–Lips/CSSH were demulsified with ethanol before and after centrifugation. Each solution was examined using UV spectrophotometry. The encapsulation efficiency (EE) and loading efficiency (DL) of Cur were calculated according to the following equations:

$$EE=\frac{c}{c^{0}}\times 100\%,$$ (2.1)

where *c* ^*0*^ and *c* represent the drug concentration before and after centrifugation, respectively.

$$DL=\frac{weight\;of\;encapsulated\;Cur}{weight\;of\;Lips}\times 100\%.$$ (2.2)

#### 2.3.3 Characterization of the drug-loaded CSSH hydrogels

To test the physical and chemical properties of the gels, the samples were divided into four groups: CSSH gels, Cur–Lip/CSSH gels (Cur concentrations of 100, 150, and 200 μM), Dox/CSSH gels (Dox concentrations of 50, 75, and 100 μM), and Cur–Lip/Dox/CSSH gels (the Cur and Dox concentrations were both 100 μM). The gelation time of each of the gels was determined using vial inversion and rheometry (Kinexus Pro, United Kingdom). Briefly, 2 ml of each of the gel precursors was placed in a serum bottle at 37°C, and the gelation time was measured and recorded. One milliliter of the gel precursor solution was placed on the sample stage of a rotating rheometer (Ø = 20 mm) followed by a time sweep test (strain = 1%, ƒ = 1 Hz, T = 37°C). The gelation time was determined as the time at which tan(*δ*) = 1.

The mechanical properties of the hydrogels were examined using a universal testing machine (Shimadzu AG-1, Japan). Each sample was placed on the sample holder, and the compression test was performed (*ν* = 2 mm/min, strain = 60%) to generate a stress‒strain curve. 1 ml of the prepared samples were placed in a dialysis bag, and then the dialysis bag was placed in a 50-ml centrifuge tube. Next, 30 ml of 2% Tween-80/PBS (pH = 7.4 and 5.5) was added to the centrifuge tube as the release medium. The tubes were placed on a shaker (T = 37°C, 100 rpm), and all the release media were removed and replaced with fresh release media after a certain period of time. All of the release media were analyzed using UV spectrophotometry, and the release rates of Cur and Dox were calculated according to the measured absorbance.

### 2.4 Cytocompatibility *in vitro*

#### 2.4.1 Cell seeding and culture

MCF-7 cells were obtained from the ATCC. Cells were cultured in Dulbecco’s modified Eagle’s medium containing 10% (v/v) fetal bovine serum and 100 U/mL penicillin‒streptomycin in a humidified atmosphere (37°C, 5% CO_2_).

#### 2.4.2 Cell proliferation and cytotoxicity

MCF-7 cells were cultured in nonessential medium containing 10% fetal bovine serum, 100 U/mL penicillin, and 0.1 mg/ml streptomycin in a humidified environment with 5% CO_2_ at 37°C. Confluent cells were trypsinized (0.25% trypsin-EDTA), centrifuged, resuspended, and counted. Briefly, the cells were seeded in the samples at a density of 3 × 10^4^ for 3 h to allow attachment, and 500 μL of the essential medium was added to each well. Cell viability was detected using a CCK-8 Kit, acridine orange/ethidium bromide (AO/EB) staining, and flow cytometry after culturing for 24, 48, and 72 h. The number of seeded cells was used as a benchmark to evaluate cell viability. The cellular uptake of drugs was determined using laser scanning confocal microscope after nuclei staining with DAPI.

Based on the Cur and Dox IC_50_ values (Supplementary Figure S1), 100, 150, and 200 μM Cur and 50, 75, and 100 μM Dox were selected for the cell experiments. There were four treatment groups in the cell experiments: Cur–Lip/CSSH gels (0, 100, 150, and 200 μM), Dox/CSSH gels (50, 75, and 100 μM), Cur–Lip/Dox/CSSH gels (0/0, 100/50, 100/75, and 100/100 μM (Cur/Dox)), and Cur–Lip/Dox/CSSH gels (0/0, 50/100, 50/150, and 50/200 μM (Cur/Dox)).

### 2.5 Statistical analysis

The data are presented as the means ± standard error (*n* = 3). The results were tested for statistical significance using SPSS. A *p* value <0.05 was considered statistically significant. Noncompartmental pharmacokinetic parameters were determined using one-phase decay analysis in GraphPad Prism v6.01 software. * indicates 0.01 < *p* < 0.05; ** indicates 0.001 < *p* < 0.01; and *** indicates *p* < 0.001.

## 3 Results and discussion

### 3.1 Analysis of CSSH

**FIGURE 2:**
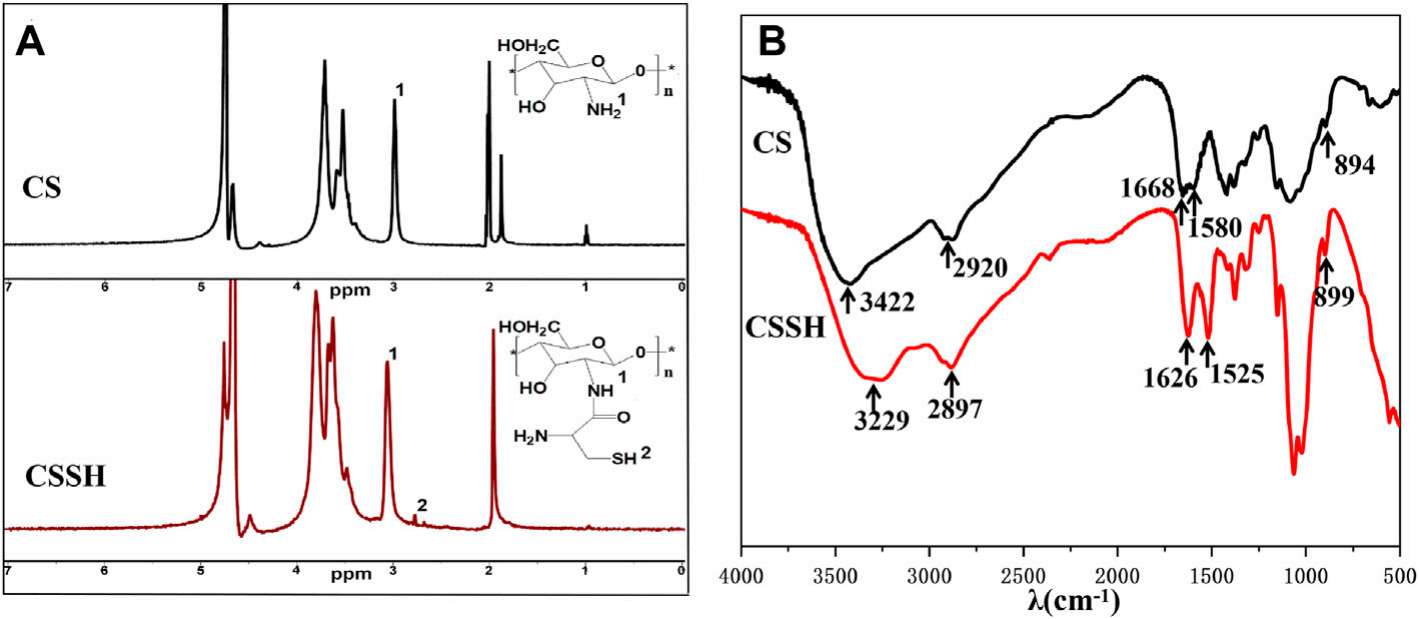
^1^H NMR spectra **(A)** and FT-IR spectra **(B)** of CS before and after modification.

The ^1^H NMR and FT-IR analysis results (Figure 2) confirmed the successful synthesis of CSSH (-SH degree of substitution was 6%, which was calculated from the ^1^H NMR peak area). The peak at *δ* = 3.01 ppm in Figure 2A represents the -NH_2_ hydrogens, and the -SH protons from CSSH were observed near *δ* = 2.7 ppm, which indicated that cysteine and -NH_2_ had combined (Hirai et al., 1991). The number of free sulfhydryl groups and total sulfhydryl groups was calculated using Ellman’s method. The degree of sulfhydryl substitution was 6%, and the contents of free and total sulfhydryl groups were 200.84 ± 16.16 μmol/g and 420.36 ± 0.14 μmol/g, respectively (Supplementary Table S1).

Compared to CS, new enhanced peaks near 1,626 cm^−1^ (-C=O) and 2,360 cm^−1^ (-SH) were observed in the FT-IR spectrum of CSSH (Figure 2B) (Brugnerotto et al., 2001; Gao et al., 2014). Combined with the ^1^H NMR results, these results confirmed the successful grafting of the sulfhydryl groups onto the CS molecular chains.

### 3.2 Characterization of the Lips

#### 3.2.1 Synthesis and analysis of the Lips

**FIGURE 3:**
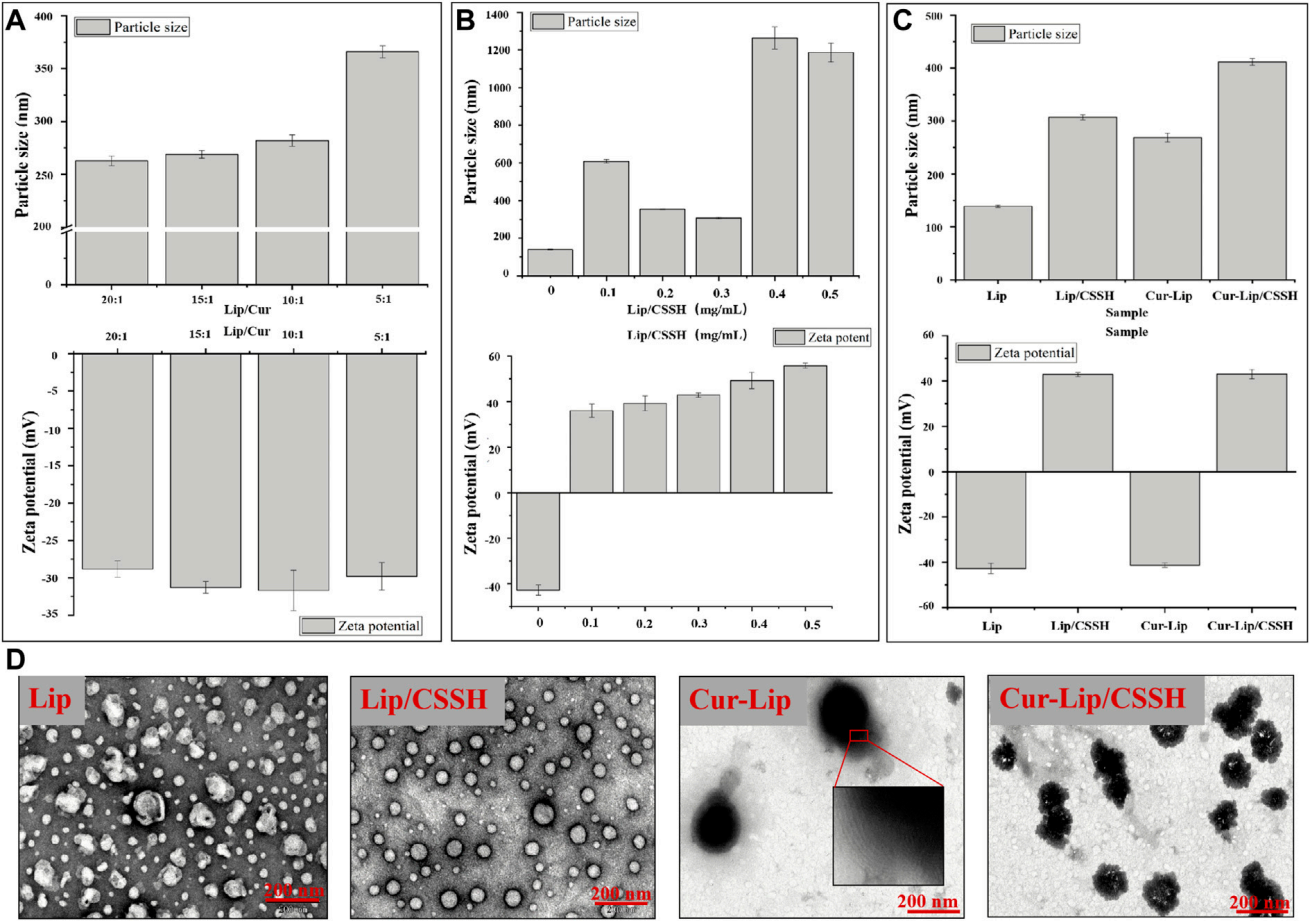
Effects of different Lip/Cur ratios on the performance of Lips **(A)**. Particle sizes and zeta potentials of the Lips after modification with different concentrations of CSSH **(B)**. Particle sizes and zeta potentials of the samples **(C)**. TEM images of the samples **(D)**.

Due to the effect of elution time on the elution efficiency of Cur and the Lips acquired using the mini column centrifugation method, we performed this procedure with the sample three times (Supplementary Table S1). The ratio of Lip to Cur (Lip/Cur) is associated with the particle size, zeta potential, and EE of the Lips. Therefore, a series of Cur-loaded Lips was designed to identify the effect of different Lip/Cur values on Lip performance. The results in Figure 3A show that the particle size gradually increased with decreasing Lip/Cur value, while the zeta potential showed no clear change. The EEs at different ratios of Lip/Cur were 72.63 ± 2.05% (20:1), 91.63 ± 2.11% (15:1), 76.84 ± 0.98% (10:1), and 57.93 ± 1.23% (5:1). Notably, the EE at Lip/Cur = 15:1 was approximately 91.63%, and the EEs at the other Lip/Cur ratios were less than 80%. Therefore, we chose a Lip/Cur ratio of 15:1 for the following studies.

The Lips were next modified by CSSH (at concentrations of 0.1, 0.2, 0.3, 0.4, and 0.5 mg/ml). The data in Supplementary Table S2 show that the particle size of Lip/CSSH exhibited a parabolic dependence on the concentration of CSSH. The smallest Lip/CSSH particle size was 307.00 ± 4.92 nm at 0.3 mg/ml CSSH. The particle size then increased rapidly to larger than 1,000 nm when the CSSH concentration was higher than 0.3 mg/ml (Figure 3B). The zeta potential of Lips/CSSH also increased from −42.8 mV to positive values with increasing CSSH concentrations. A previous study found that in A431D cells, a model cell line frequently used in carcinoma research, giant unilamellar vesicles (GUVs) with a zeta potential of −31 mV had an almost 100-fold increase in cell attraction compared to GUVs with a +2 mV zeta potential. Conversely, GUVs with a zeta potential of +28 mV had a 50-fold greater attraction than GUVs with a zeta potential of +2 mV (Staufer et al., 2020). These data suggest that a higher Lip zeta potential facilitates interactions with cells. However, the size of the Lips affects their distribution in the blood because smaller Lips are more likely to diffuse into tumors from capillaries. Therefore, a CSSH concentration of 0.3 mg/ml was selected for subsequent study.

#### 3.2.2 Morphology analysis

According to the preliminary results, samples were prepared at a CSSH concentration of 0.3 mg/ml with Lip/Cur = 15:1. The morphology, particle size, and zeta potential of the formulated Lips are shown in Figure 3. The particle sizes of the samples measured using DLS were 139.11 ± 1.78 nm (Lip), 268.73 ± 3.19 nm (Cur–Lip), 307.00 ± 4.92 nm (Lip/CSSH), and 411.83 ± 6.47 nm (Cur–Lip/CSSH) (Figure 3C). The increase in particle size was due to the Cur molecules entering the Lip bilayer and the CSSH modification on the Lip surface. The TEM images showed that the particle size of the Lips was approximately 50–200 nm (Figure 3D), which was smaller than the particle size measured using DLS (Figure 3C). This result was primarily because the Lips shrank during the freeze-drying process.

Lips not coated with CSSH showed an irregular spherical shape and aggregation, and the CSSH-coated Lips had a regular spherical structure and uniform particle size distribution (Figure 3D). These differences indicated that CSSH increased the stability of Lips. Lip fingerprint structures were observed on the Cur–Lip surface. The zeta potentials of the Lips were −42.80 ± 2.31 mV (Lip), 42.92 ± 0.94 mV (Lip/CSSH), −41.30 ± 1.01 mV (Cur–Lip), and 43.07 ± 1.99 mV (Cur–Lip/CSSH).

#### 3.2.3 Drug release behavior

**FIGURE 4:**
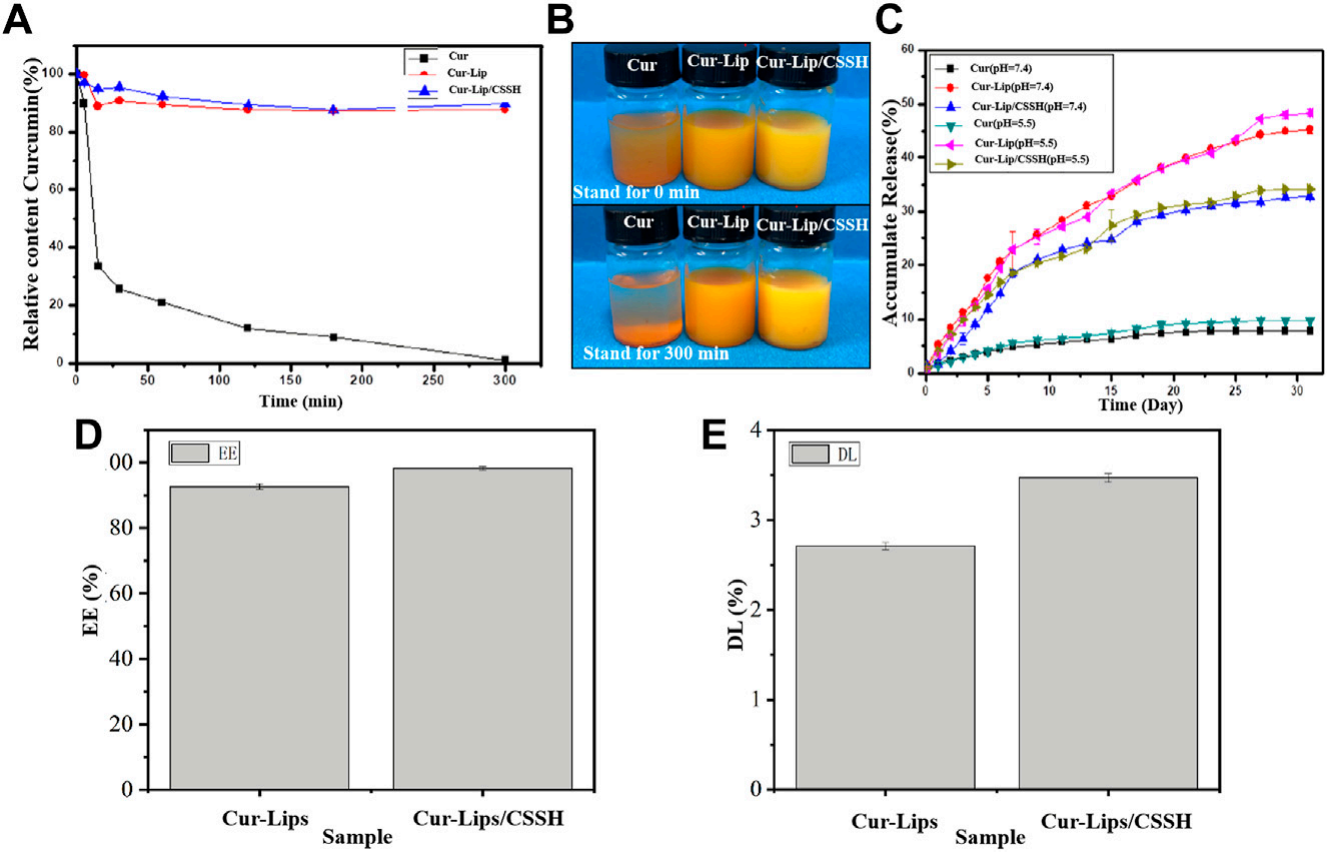
Stability of Cur in different solutions **(A,B)**. Cumulative release of Cur *in vitro* in different solutions **(C)**. EE **(D)** and DL **(E)** of the Lips.

The curve in Figure 4A shows that the concentration of Cur in the Cur solution was 32% after 15 min and was almost 0 after 300 min. However, the Cur concentration was maintained at approximately 90% in the Cur–Lip and Cur–Lip/CSSH solutions. This same phenomenon is displayed in the macro diagram in Figure 4B. Cur encapsulated into Lips functionalized with CSSH remained stable (Figure 4). The main reason for the low bioavailability of Cur is its low water solubility. However, its encapsulation into Lips effectively resolved this problem. The cumulative release curve in Figure 4C shows that the linear release of Cur in each group at different pH values was almost the same. The cumulative release rates of Cur in each group after 30 days were as follows: approximately 10% (Cur), 50% (Cur–Lip), and 30% (Cur–Lip/CSSH). These results indicated that Lips improved the water solubility and bioavailability of Cur to a large extent.

As shown in Figures 4D,E, the EE and DL of the Cur–Lips were 92.63 ± 0.87% and 2.71 ± 0.04%, respectively (the Cur standard curve is shown in Supplementary Figure S1). These values increased to 98.23 ± 0.48% (EE) and 3.47 ± 0.05% (DL) after CSSH modification. This increase occurred because the Lips encapsulated Cur with its hydrophobic head, and the mobility of the bimolecular layer increased the likelihood of drug release. After modification with CSSH, a “coat” was formed *via* electrostatic attraction between CSSH and Cur, which limited the mobility of the Lip bimolecular layer and reduced the liberation of Cur. These results indicated that the water solubility of Cur affected its bioavailability and the stability of the solution.

#### 3.2.4 Cytotoxicity of the lips

**FIGURE 5:**
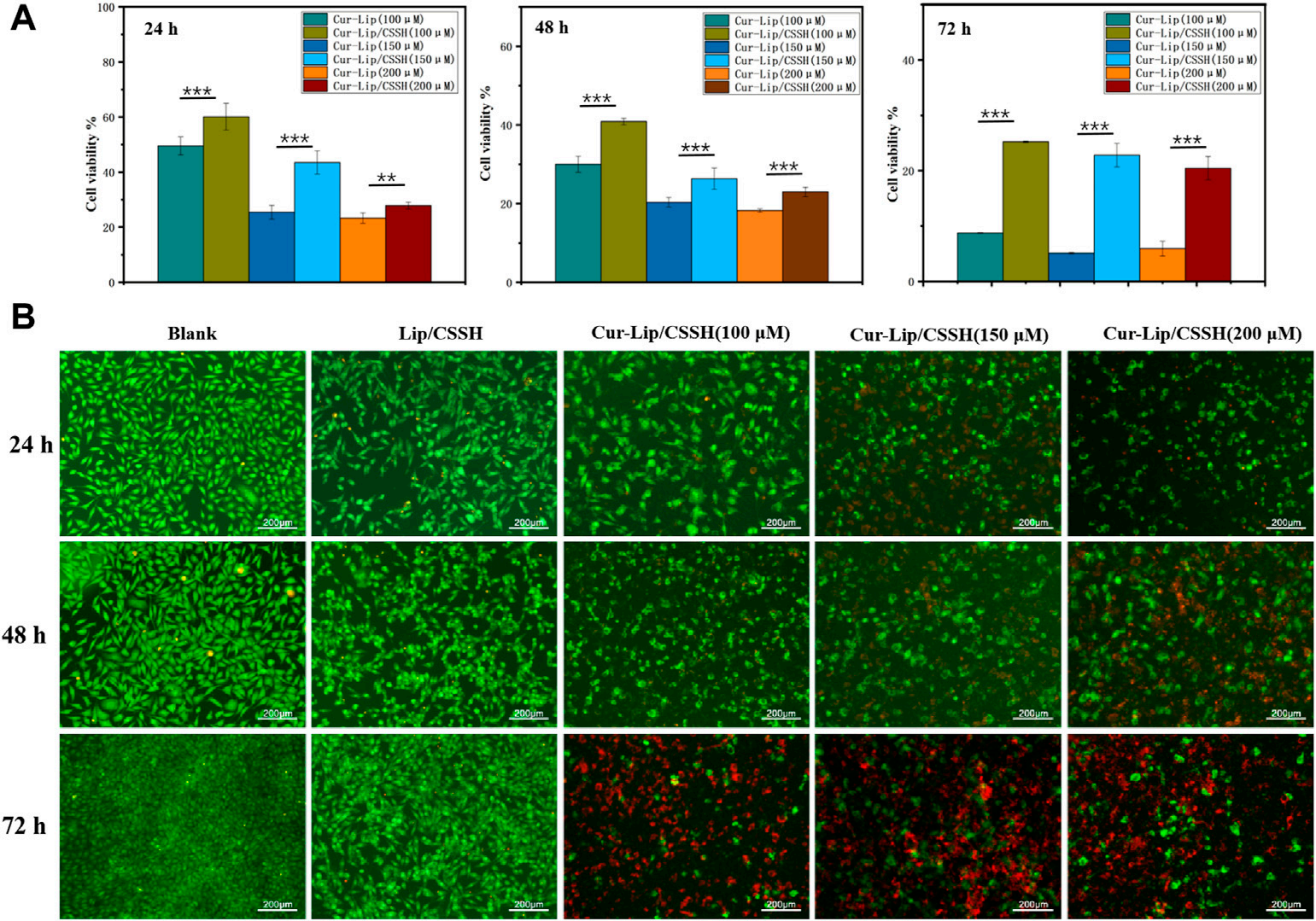
Cell proliferation **(A)** and AO/BE staining image **(B)**.

According to the Cur IC_50_ results (Supplementary Figure S1), Cur–Lips and Cur–Lips/CSSH with Cur concentrations of 100 μM, 150 μM, and 200 μM were selected for cell assays. The results showed that all samples exhibited drug concentration-dependent cytotoxicity (Figure 5). Cell viability after treatment with Cur–Lips/CSSH was significantly higher than after the administration of Cur–Lips (Figure 5A). This result occurred because the release of Cur from Lips-CSSH was slower than that of pure Lips. Similar results were obtained after live/dead staining, as shown in the photomicrograph in Figure 5B (AO/BE staining; green indicates live cells and red indicates dead cells), which indicated that the addition of CSSH did not cause significant cytotoxicity, but good biocompatibility was retained.

### 3.3 Characterization of the hydrogels

#### 3.3.1 Physical and chemical properties of the drug-loaded gels

**FIGURE 6:**
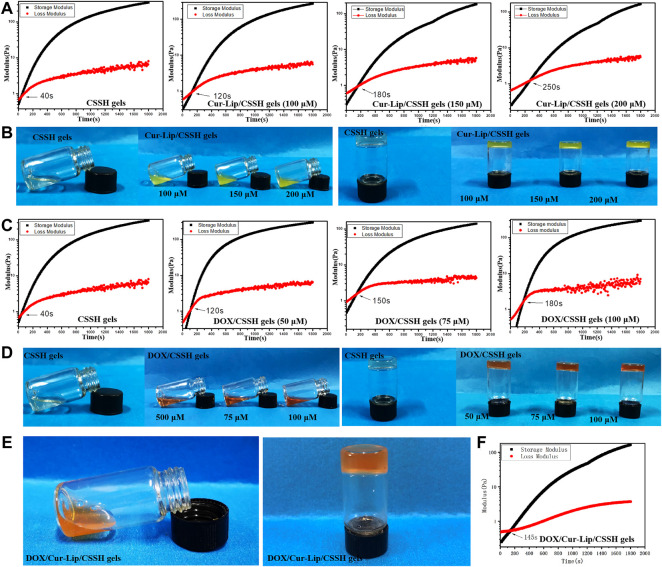
Gelation time and gelation point of the drug-loaded hydrogels measured using vial inversion **(B,D,E)** and rheology analysis **(A,C,F)**.

The results in Figure 6 show that gelation at 37°C was completed within 8∼12 min. However, the gelation point shifted to longer times with the addition of the drug. This shift presumably occurred because the surface of the Cur–Lips was negatively charged and prone to electrostatic interactions with the positively charged CSSH. Therefore, the interaction between the sulfhydryl groups was weakened and became more significant with increasing concentrations of Lips.

#### 3.3.2 Mechanical and drug release analysis

**FIGURE 7:**
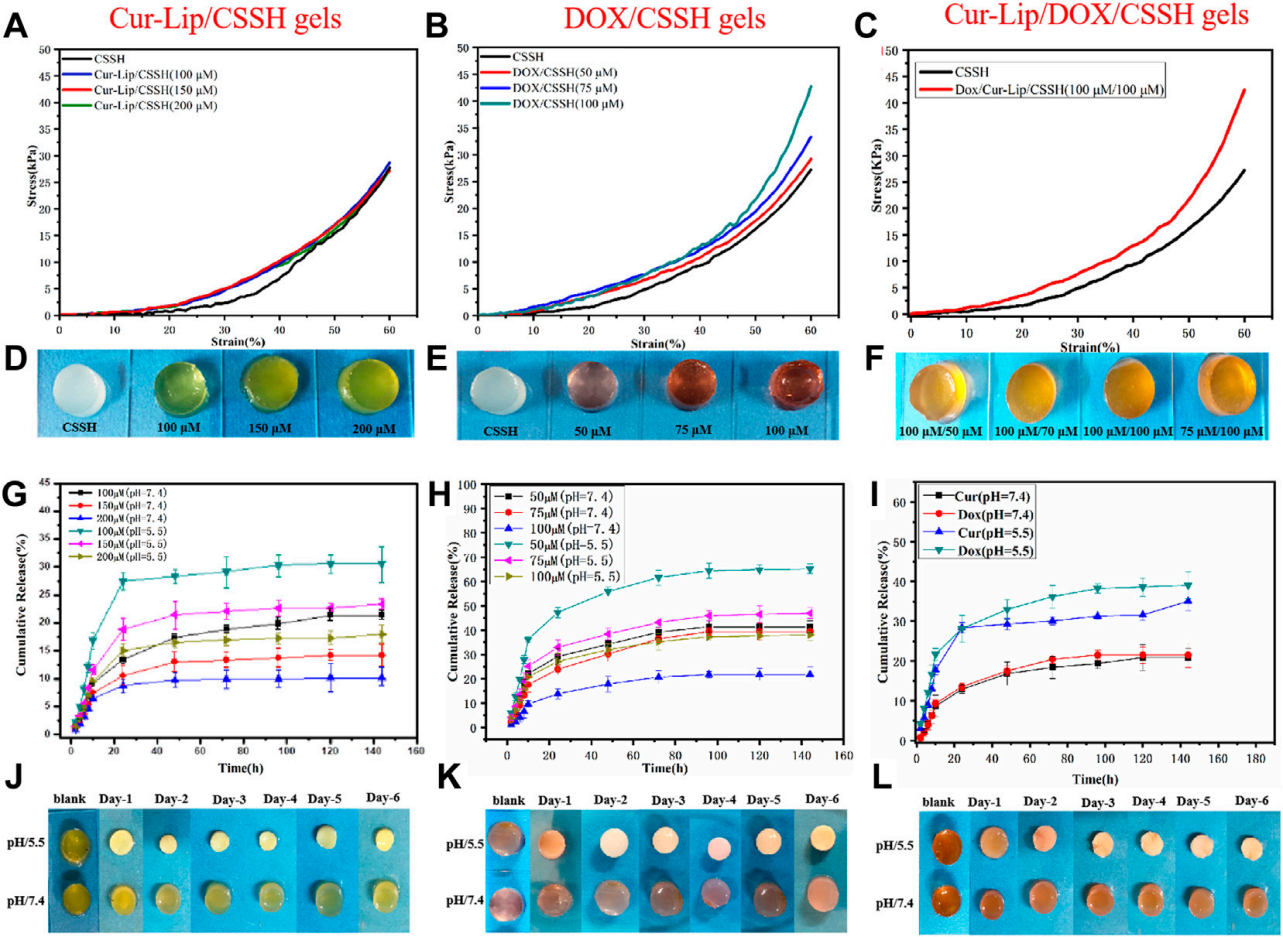
Stress‒strain curves of the gels **(A–C)**. Drug release curves of the gels containing different concentrations of drug **(G,H)**. Drug release curve of the Cur–Lip/Dox/CSSH gels (100 µM/100 µM). **(I)** Macro images of the gels **(D–F,J–L)**.

The compression stress‒strain curves are shown in Figures 7A–C. The compressive strength of the Cur–Lip/CSSH gels ranged from 25–30 kPa and was independent of the drug concentration. The compressive modulus of the Cur–Lip/CSSH gels was higher than that of the CSSH gels, which was due to the increase in the cross-linking density of the gels after the addition of Cur–Lips. The Dox/CSSH gels showed a dose dependency, and the highest stress was 43 kPa. These results may be due to the abundant -OH groups present in the Dox structure, which form hydrogen bonds with the CSSH chains. This same trend was evident in the mechanical properties of the Dox/Cur–Lip/CSSH gels (Figures 7G–I).

*In vitro* drug release experiments reflect drug bioavailability to a certain extent [Figures 7G–I, and the standard curve of Dox release into medium (Supplementary Figures S2, S3)]. As shown in the cumulative release curve in Figure 7, Cur and Dox were rapidly released within 24 h. Notably, the cumulative drug release increased almost linearly over the first 10 h. The cumulative release of Cur (100 µM) was 27.14% in the single-drug systems, and Dox (50 µM) release was 47.27% after 24 h at pH = 5.5. After the first 24 h, the release of Cur slowed due to encapsulation by the gel. In contrast, Dox maintained a steady release rate because Dox is water-soluble and contains a large number of -OH groups that form hydrogen bonds with CS. This property promoted the slow and long-lasting release of Dox. The drugs exhibited higher release at pH = 5.5. This result was primarily caused by the increased swelling of the CSSH gels under acidic conditions. The addition of Cur–Lips/Dox should make the CSSH gel networks denser. This increased density slows the diffusion of the drug molecules, which is reflected by the lower release at higher concentrations. Figure 7I shows drug release from the binary drug system at pH 5.5 and 7.4. The results indicated that approximately 15% of the drugs were released within the first 3 days, after which no further decrease in the residual drug was observed at pH = 7.4. However, the release of Cur and Dox was approximately 30% during the first 24 h, and the Dox release was approximately 10% from 24 to 120 h at pH = 5.5, which favored cancer cell death. These data indicated that drug release from the Cur–Lip/Dox/CSSH gels was highly correlated with pH, which is a key point for the application of Cur–Lips/Dox/CSSH in the acidic tumor microenvironment.

### 3.4 Cytotoxicity of the hydrogels

**FIGURE 8:**
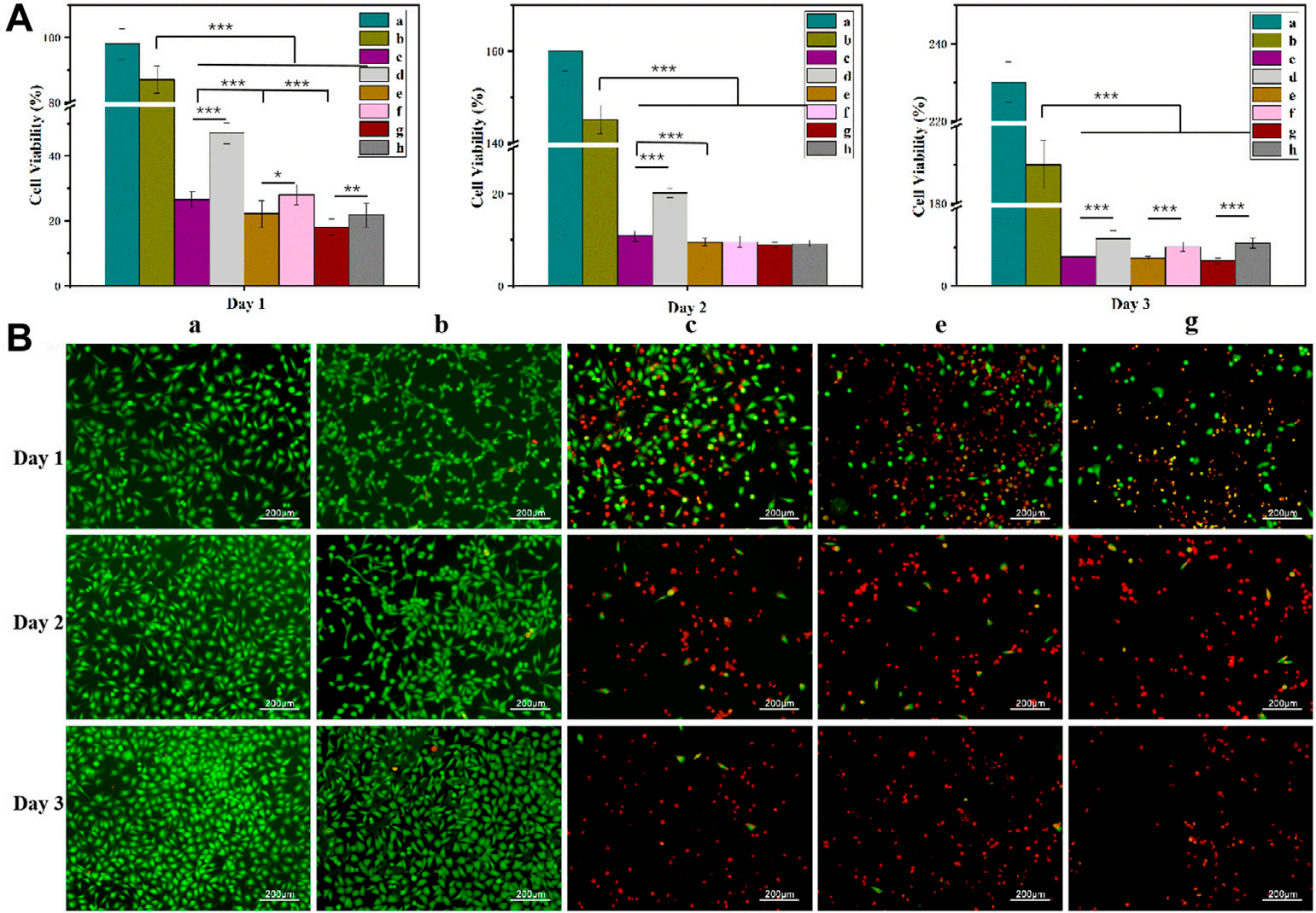
MCF-7 cell viability measured using the CCK-8 assay **(A)** and AO/EB staining results **(B)**. a: Blank; b: CSSH gels; c: Cur–Lip/Dox/CSSH gels (100 μM/50 μM Cur/Dox); d: Dox/CSSH gels (50 μM); e: Cur–Lip/Dox/CSSH gels (100 μM/75 μM Cur/Dox); f: Dox/CSSH gels (75 μM); g: Cur–Lip/Dox/CSSH gels (100 μM/100 μM Cur/Dox); and h: Dox/CSSH gels (100 μM).

**FIGURE 9:**
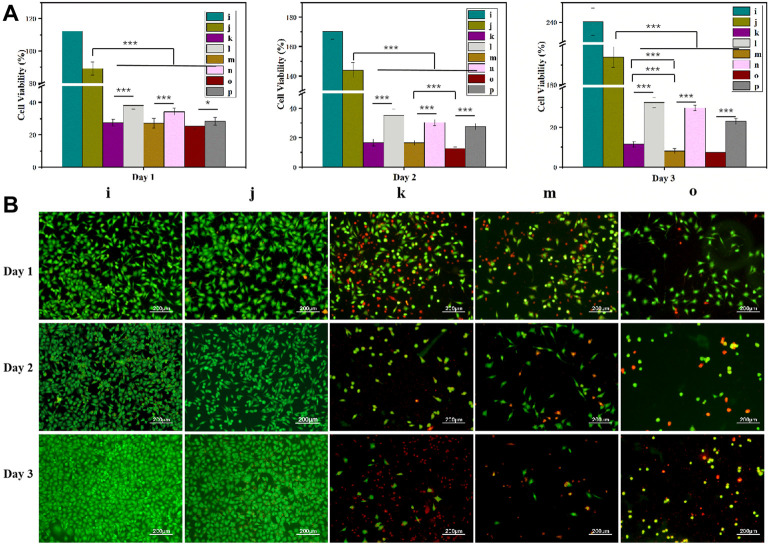
MCF-7 cell viability measured using the CCK-8 assay **(A)** and AO/EB staining **(B)**. i: Blank; j: CSSH gels k: Cur–Lip/Dox/CSSH gels (100 μM/50 μM Cur/Dox); l: Cur–Lip/CSSH gels (100 μM); m: Cur–Lip/Dox/CSSH gels (150 μM/50 μM Cur/Dox); n: Cur–Lip/CSSH gels (150 μM); o: Cur–Lip/Dox/CSSH gels (200 μM/50 μM Cur/Dox); and p: Cur–Lip/CSSH gels (200 μM).

Based on the Cur and Dox IC_50_ results (Supplementary Figure S1), the selected drug concentrations for all experiments were 100, 150, and 200 μM (Cur) and 50, 75, and 100 μM (Dox) for the experiments. All gels loaded with drugs showed concentration-dependent cytotoxicity (Figures 5, 8, 9). These results found that cell viability after treatment with the single drug-loaded CSSH gels was significantly higher than that in cells administered the dual drug-loaded CSSH gels. Blank CSSH gels consistently showed good biocompatibility with MCF-7 cells.

These results indicated that cell viability after treatment with the Cur–Lip/CSSH gels was significantly higher than after Cur–Lip/Dox/CSSH gel administration. The AO/EB staining images in Figure 8B (where green indicates live cells and red indicates dead cells) supported this conclusion. At the same concentration of Dox, the viability of cells cultured with different concentrations of Cur–Lips was 26.51%, 22.13%, and 18.10% (Day 1); 10.82%, 9.45%, and 8.87% (Day 2); and 7.51%, 7.22%, and 6.45% (Day 3). Therefore, changes in the Cur–Lip concentration did not have a significant effect on cell viability. No significant difference in drug release was observed during the initial release stage when comparing the single- and dual drug-loaded CSSH gels (Figure 8). However, the significant differences found between the single- and dual drug-loaded gels after 3 days of culture indicated that the slow release of Cur from the Lips effectively inhibited the growth of cancer cells.

After culturing on drug-loaded CSSH gels for 1 day (Figure 9A), the viabilities of MCF-7 cells were 27.50%, 27.03%, and 25.35% (dual drug-loaded) and 38.11%, 34.21%, and 28.32% (single drug-loaded). Over time, cell viability decreased to 11.52%, 8.07%, and 7.55% (double drug-loaded) and 32.56%, 29.81%, and 23.10% (single drug-loaded) on day 3 (Figure 9A). The single- and dual drug-loaded CSSH gels containing different concentrations of Cur–Lips showed significant differences in their abilities to inhibit the growth of cancer cells. However, cell viability decreased with increasing Cur–Lip concentration. Cur increases mitochondrial membrane permeability, which results in an increase in membrane potential and a loss in the ability to bind ATP (Morin et al., 2001). However, Dox is a well-known traditional broad-spectrum antineoplastic drug (Chan et al., 1999; Cui et al., 2019). Overall, the inhibitory effect of the combination of Cur–Lips with Dox on MCF-7 cells was more significant.

**FIGURE 10:**
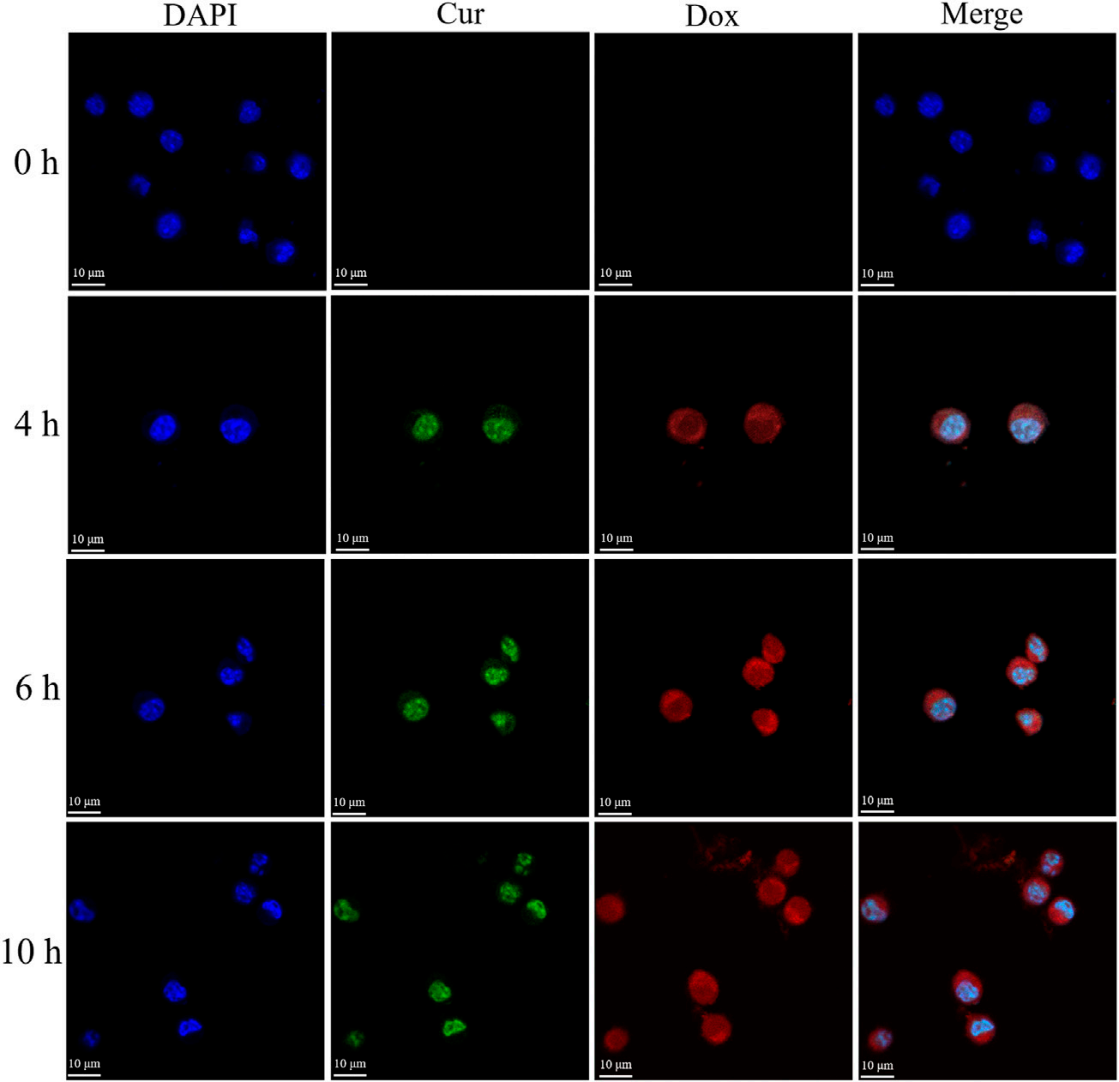
Laser scanning confocal microscopy (LSCM) images of MCF-7 cells.

Figure 10 shows the uptake of Cur and Dox by MCF-7 cells based on the measured spontaneous green fluorescence of Cur and spontaneous red fluorescence of Dox. Over time, the fluorescence intensity in the nuclei of the MCF-7 cells increased. These results indicated that more Cur and Dox were taken up by cells over time, which inhibited their proliferation.

## 4 Conclusion

The current study prepared and characterized a series of Cur–Lip/Dox/CSSH gels. The results showed that the CSSH gels possessed good, rapid *in situ* gelation and mechanical properties. The *in vitro* drug release results demonstrated greater drug release from the Cur–Lip/Dox/CSSH gels in an acidic environment that was similar to the acidic environment in tumors. The cell culture assays showed that the dual drug-loaded gels exhibited superior inhibitory effects on MCF-7 cells compared to the single drug-loaded gels. This result indicated that the inclusion of two drugs into this release system produced synergy when in coculture. The present study validated the advantages of *in situ*-formed injectable hydrogels as drug carriers and their potential applications for the treatment of breast cancer. However, the mechanism underlying the inhibitory effect of Cur combined with Dox on MCF-7 cells is not clear and requires further experiments.

## Data Availability

The original contributions presented in the study are included in the article/Supplementary Material; further inquiries can be directed to the corresponding authors.
