# Supplementary material for: Temperature- and pH-responsive injectable chitosan hydrogels loaded with doxorubicin and curcumin as long-lasting release platforms for the treatment of solid tumors
Source: Front Bioeng Biotechnol. 2022 Nov 3;10:1043939. doi: 10.3389/fbioe.2022.1043939 (PMC9669971; doi:10.3389/fbioe.2022.1043939)
Supplement: Supplementary file 1 [file DataSheet1.docx]

**1.1 The existence of –SH analysised by Fourier infrared spectrometer (FTIR, Quinox 55, Germany) and Nuclear Magnetic Resonance Spectroscopy.**

The existence of –SH was confirmed by Fourier infrared spectrometer (FTIR, Quinox 55, Germany) and Nuclear Magnetic Resonance Spectroscopy (^1^H NMR, AVANCE III 500, Germany). Briefly, the samples mixed with KBr were pressed into a flaky shape. 5 mg of CS and CSSH samples were added into a centrifuge tube, then 0.55 mL D_2_O/CF_3_COOD (95:5 v/v) and 0.55 mL D_2_O were added respectively. The resulting mixed solution was placed in a nuclear magnetic tube for ^1^H NMR analysis (AVANCE III 500 Germany).

**1.2 The encapsulation efficiency (EE) and loading efficiency (DL) of Cur-Lips**

The Cur-Lip and Cur-Lip/CSSH before and after centrifugation were demulsified with ethanol. Then, the solution was measured by UV spectrophotometer. The encapsulation efficiency (EE) and loading efficiency (DL) of Cur were calculated according to the following equations.

$EE=\frac{c}{c^{0}}\times100\%$ （2.1）

Where *c^0^* and *c* stand for the drug concentration before and after centrifugation, respectively.

$DL=\frac{weight of encapsulated Cur}{weight of Lip}\times100\%$ （2.2）

**1.3 The gelation time, gel point time and mechanical properties of the hydrogels**

The gelation time of gels were confirmed by the *vial* inversion and rheometer (Kinexus Pro, UK). Briefly, 2 mL of gel precursors were placed in serum bottle and stored in water bath environment at 37 °C. Once the liquid changed to solid, the *vials* were removed and the gelation time was recorded. 1 mL of gel precursor solution was placed on the sample stage of the rotating rheometer (Ø=20 mm), then a strain scanning was performed on the gel precursor samples (strain=1%, ƒ=1 Hz, T=37 °C). The gel point time was determined by the intersection of the storage modulus (G´) and the loss modulus (G´´).

The mechanical properties of the hydrogels were confirmed by Universal testing machine (SHMADZU AG-1, Japan). The samples were placed on the sample table, and the compression test was carried out (ν = 2 mm/min, strain = 60%) to obtain a stress-strain curve.

The prepared hydrogel samples were placed in a 50 mL centrifuge tubes, then 30 mL of 2% Tween-80/PBS (pH = 7.4/5.5) was added. Next, the tubes were put on a shaker (T = 37 °C，100 rpm), all the release mediums were analyzed by UV spectrophotometer and replace with fresh release medium at preset time. The Cur and Dox release was calculate according to the absorbance of release medium and drug/medium standard curve.

Table 1 The Degree of Substitution of Chitosan Derivative, Free Thiol Group and Total Thiol Group of CSSH

| DS* | -SH(free)^**^(μmol/g） | -SH(sum)^**^^*^(μmol/g） |
| --- | --- | --- |
| 6% | 200.84±16.16 | 420.36±0.14 |

* The degree of sulfhydryl substitution calculated by ^1^H NMR

** the content of free sulfhydryl groups（A = 3.8981C - 0.0312，R^2^ = 0.9995）

*** the content of total sulfhydryl groups（A = 0.8235C + 0.0045，R^2^ = 0.9997）

Table 2 The Effect of elution times on elution efficiency

| elution times | 1 | 2 | 3 | 4 | 5 |
| --- | --- | --- | --- | --- | --- |
| Cur | 0.00% | 0.00% | 0.00% | 0.44% | 0.46% |
| Lip | 65.45% | 94.55% | 99.80% | 99.98% | 99.98% |
| Cur + Lip | 1.91% | 2.90% | 3.82% | 3.82% | 3.82% |

The standard curve of Cur/ethanol: A=0.16683c-0.01587，R^2^=0.9995

Tab.2 showed the effect of elution times on the elution efficiency of Cur and Lips measured by mini column centrifugation method. The results indicated that the dextran gel mini column exhibited excellent adsorption effect on free Cur and almost the greatest adsorption efficiency on blank Lips after eluted for three times. However, the elution efficiency of Cur in the mixture of Cur + Lip was 3.82% which was a lot bigger than the free Cur. This was because a small amount of Cur entered the bilayer of Lips in the process of mixing Lips and free Cur and be eluted together with the Lips. Therefore, the final selection of elution times was determined to be 3 according to the experimental results.


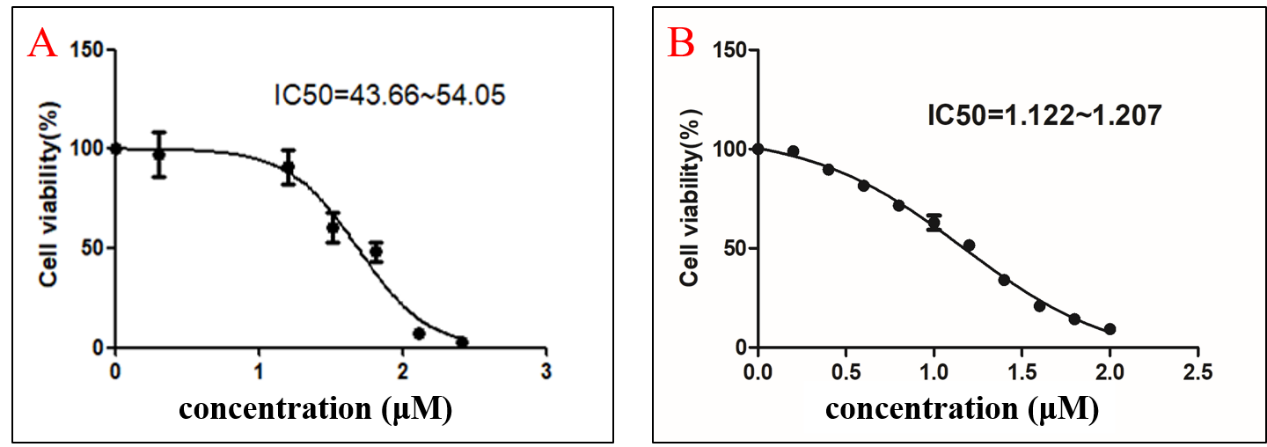


Fig.1 The IC_50_ of MCF-7 cells dealed with Cur (A) and Dox (B)

In vitro release of Cur was investigated in PBS containing 2% (v/v) Tween-80 (release medium) by UV spectrophotometer. Briefly, 10 mg Cur was resolved in 10 mL deionized water to homogenize Cur distribution by ultrasonic oscillation as the blank group, then 1 mL of Cur-Lips/CSSH solution obtained as described above were encapsulated into a dialysis bag (8000 Da) respectively. Next, the dialysis bag was added into a breaker with 50 mL of release medium in it (pH = 7.4/5.5). The experiment was performed in triplicate at 37 °C with shaking at 100 rpm. All samples were withdrawn at preset time intervals and replaced with refresh release medium. The absorbance of release mediums was analyzed by UV spectrophotometer (UV-2550, Japan) and the release of Cur was calculated according the absorbance and Cur/release medium standard curve. In vitro release of Dox was investigated in the same way as Cur.


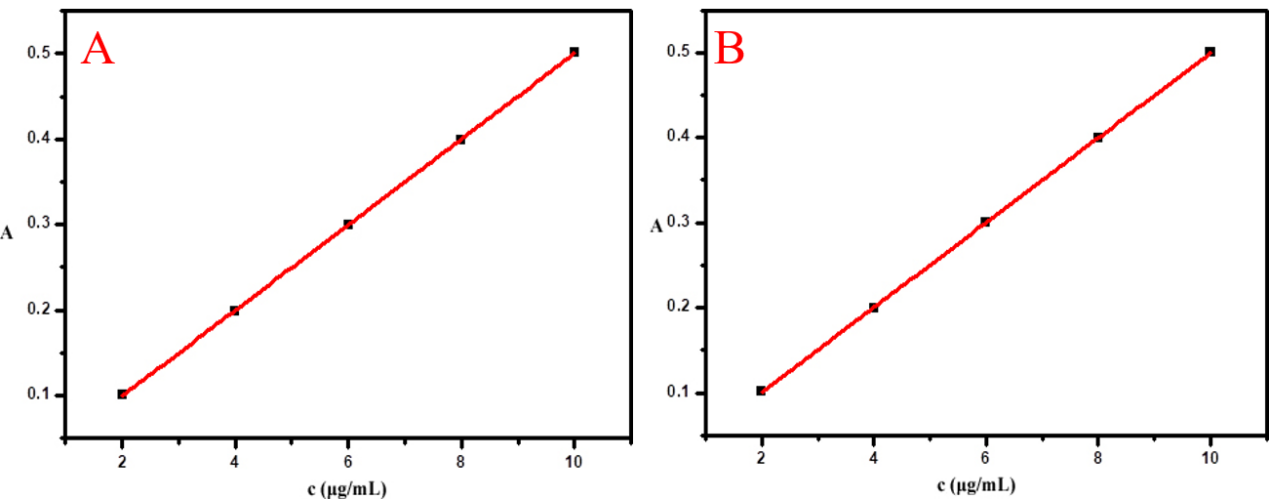


Fig.2 The standard curve of curcumin resolved in release medium of pH=7.4 (A) and 5.5 (B)

The standard curve of Cur in release medium of pH=7.4: A=0.05007*c-5.3333*10^-4^, R^2^=0.99995

The standard curve of Cur in release medium of pH=5.5: A=0.04987*c-8.0000*10^-4^, R^2^=0.99994


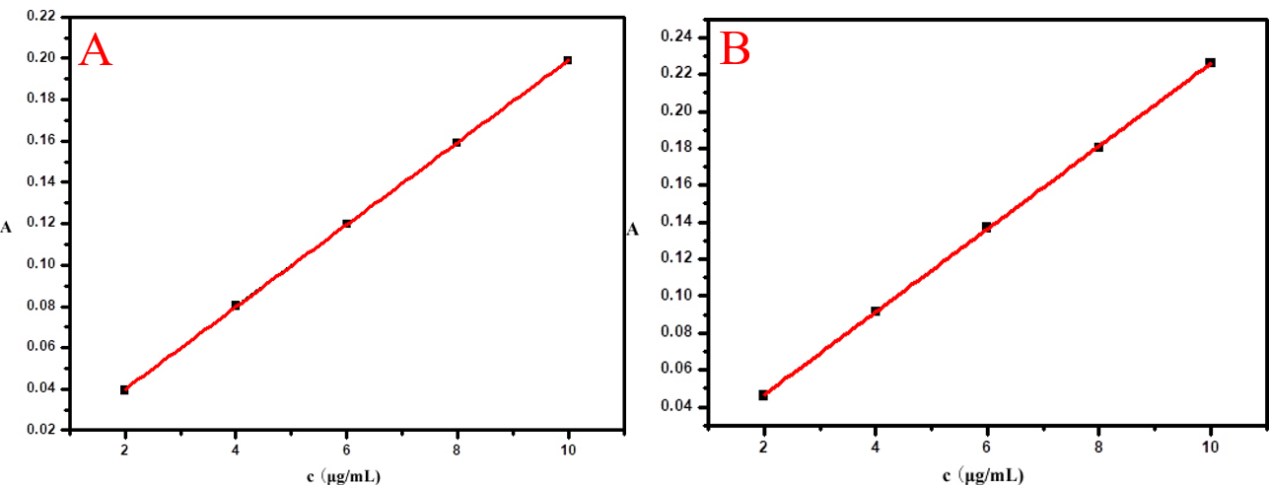


Fig.3 The standard curve of Dox resolved in release medium of pH=7.4 (A) and 5.5 (B)

The standard curve of Cur in release medium of pH=7.4: A=0.01990*c-6.6667*10^-5^, R^2^=0.99993

The standard curve of Cur in release medium of pH=5.5: A=0.02243*c-0.0016*10^-5^, R^2^=0.99994


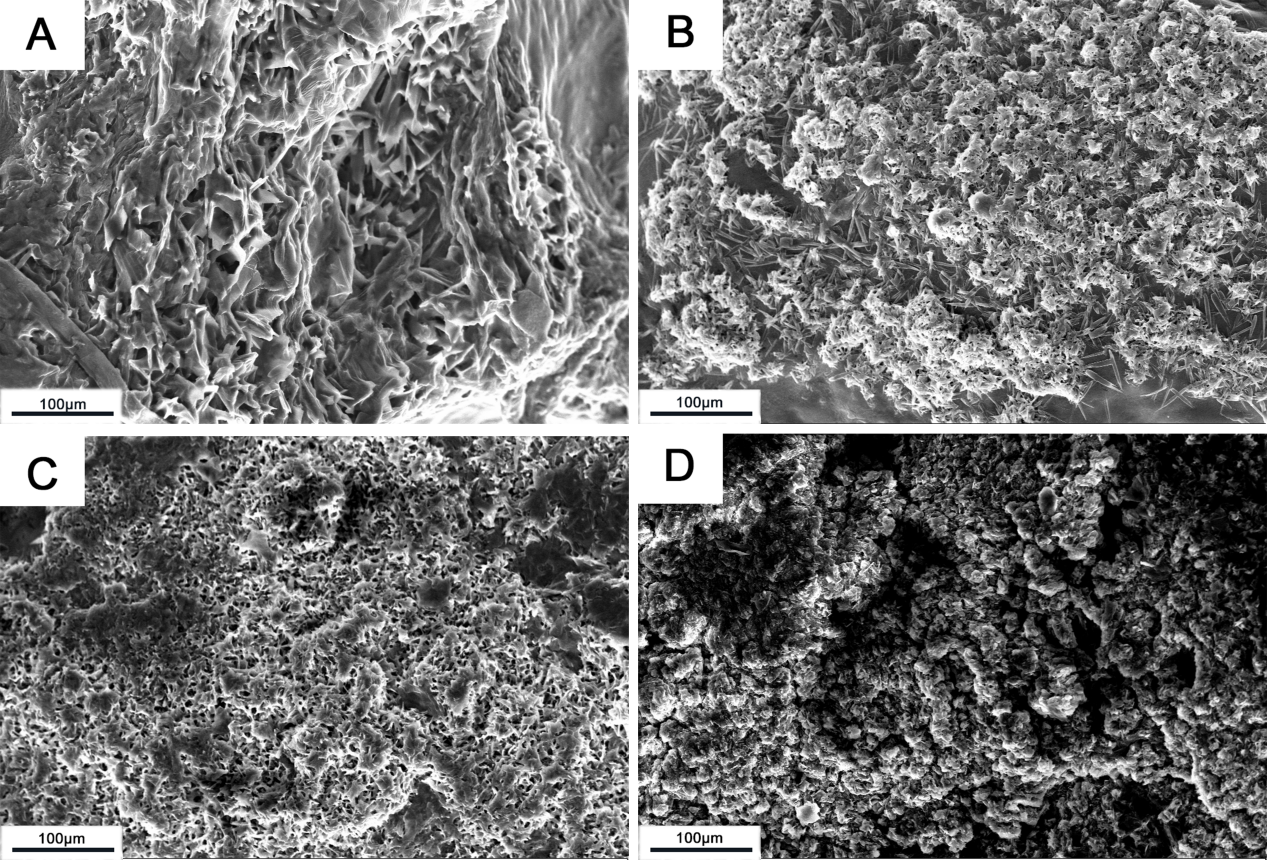


Fig4. the SEM Graphs of Hydrogel: Blank Hydrogels(A), 50 μM Doxorubicin Concentration of Dox Hydrogels(B), 75 μM Doxorubicin Concentration of Dox Hydrogels(C) and 100 μM Doxorubicin Concentration of Dox Hydrogels(D)
